# Hydroxychloroquine, TTP, COVID-19, and SLE

**DOI:** 10.4274/tjh.galenos.2021.2020.0770

**Published:** 2021-02-25

**Authors:** Pathum Sookaromdee, Viroj Wiwanitkit

**Affiliations:** 1 Private Academic Consultant, Bangkok, Thailand; 2 Adjunct Professor, Joseph Ayobabalola University, Ikeji-Arakeji, Nigeria

**Keywords:** Hydroxychloroquine, TTP, COVID-19, SLE

## To the Editor,

The recent report entitled “Hydroxychloroquine-associated thrombotic thrombocytopenic purpura” (TTP) was very interesting [1]. Arıkan et al. [1] concluded that the patient represented a possible case of hydroxychloroquine-induced TTP and called for awareness of this possible adverse effect of hydroxychloroquine (HQ) in alternative therapy for coronavirus disease-19 (COVID-19). In fact, TTP might be the result of many disorders. In the present case, it can be confirmed that the patient had TTP, but the etiology is not clear. There is a lack of explanation of the patient’s presenting symptom. The exact disease of the patient at the first presentation is a question to be discussed.

First, hydroxychloroquine-related TTP is extremely rare and the dosage should be high. In this patient, the dosage might not have been high. Also, there are many criteria of the Naranjo scale that might not have been completely fulfilled, such as proven drug existence in the blood, history of previous reaction, reappearance after re-administration, worsening after increasing dose, improvement after discontinuation, and exclusion of other possible objective evidence. Second, whether this was COVID-19 or not has to be discussed. It is clear that there was a negative polymerase chain reaction (PCR) result. It is possible that this patient might have had a clinical presentation resembling a COVID-19 case such that the physician in charge decided to use H alternative therapy without waiting for the PCR test for COVID-19. Regarding negative PCR results, there is a chance of a false negative. In a recent publication, Arevalo-Rodriguez et al. [2] stressed “the need for repeated testing in patients with suspicion of SARS-CoV-2 infection given that up to 54% of COVID-19 patients may have an initial false-negative reverse transcription polymerase chain reaction.” To decrease the false negative rate, it was suggested to consider the evidence of abnormal blood aspartate aminotransferase and lactate dehydrogenase level [3], which were also observed in the present case. Additionally, TTP might also be induced by COVID-19 [4].

Third, if we believe that this was not a COVID-19 case, it should be further considered what illness the patient had. Another possibility that might be easily forgotten is systemic lupus erythematosus (SLE). The patient had many clinical features, such as hematological findings, that might be seen in SLE. Nevertheless, there was a lack of complete laboratory work-up for SLE; therefore, that diagnosis was not possible. In SLE, TTP might be the clinical presentation [5]. The use of steroids as well as plasmapheresis can also help treat patients with SLE-related TTP.
