# Supplementary material for: Hydroxychloroquine, TTP, COVID-19, and SLE
Source: Turk J Haematol. 2021 Feb 25;38(1):99–100. doi: 10.4274/tjh.galenos.2021.2020.0770 (PMC7927449; doi:10.4274/tjh.galenos.2021.2020.0770)
Supplement: Related texts

# Reply

## To the Editor,

We thank our colleagues for their valuable comments and interest in our recently published letter [1]. Thrombotic thrombocytopenic purpura (TTP) is a life-threatening thrombotic microangiopathy. Most patients present with a pentad of symptoms. However, only less than 10% experience all elements of the identified criteria. Most of the symptoms related to TTP are caused by microthrombi [2]. It is quite possible to have patients who present with symptoms attributable to tissue/organ ischemia. Thus, chest/back pain as the first presenting symptom of TTP has been reported, and after exclusion of other life-threatening conditions, it should be considered as a disease-related symptom as well [3,4]. We did not mention that this symptom is regarded as a presenting symptom of TTP, since the manuscript length is restricted for letters. The patient had two negative polymerase chain reaction (PCR) results for COVID-19 and chest computed tomography (CT) with angiography did not reveal pulmonary thromboembolism. Cardiac markers were not consistent with acute coronary syndrome. Chest/back pain was relieved by plasma exchange therapy. Thus, the first presenting symptom of the patient was clearly related to TTP.

We presented the whole Naranjo scale and calculated an overall score, according to which we declared that a relationship between hydroxychloroquine (HQ) and TTP was possible. We did not claim that all Naranjo criteria were fulfilled. Moreover, there is no need to have a full Naranjo score to draw a causal relationship.

COVID-19 has persisted for about a year or so. Information about the clinical symptoms, diagnosis, and definite treatment is still scarce. Almost all treatment options initially accepted as the gold standard have been substantially changed by the results of randomized controlled trials. Thus, all we could do was perform PCR and thorax CT to make a diagnosis. We did so, and we had negative results twice. What more could have we done to exclude the possibility of COVID-19? Further discussion would be mere speculation. However, people do die because of the catastrophic course of this disease, and we think that COVID-19 leading to TTP would have followed a more catastrophic clinical course than that experienced by our patient.

Finally, most cases of TTP are idiopathic. In our case, however, we have claimed a possible relationship between HQ and TTP. Systemic lupus erythematosus can be an underlying disease of TTP. However, it is mostly seen in women of child-bearing age; it is extremely rare in 65-year-old men.

Best Regards,

**Fatma Arıkan, Yasin Yıldız, Tarık Ercan, Özen Oruç, Seçkin Akçay, Fergun Yılmaz, Tayfur Toptaş, Tülin Tuğlular**

## References

[1] Arıkan F, Yıldız Y, Ercan T, Oruç O, Akçay S, Yılmaz F, Toptaş T, Tuğlular T (2020). Hydroxychloroquine-associated thrombotic thrombocytopenic purpura. Turk J Hematol.

[2] Arıkan F, Yıldız Y, Ercan T, Oruç O, Akçay S, Yılmaz F, Toptaş T, Tuğlular T (2020). Hydroxychloroquine-associated thrombotic thrombocytopenic purpura. Turk J Hematol.

[3] Griffin D, Al-Nouri ZL, Muthurajah D, Ross JR, Ballard RB, Terrell DR, Vesely SK, George JN, Marques MB (2013). First symptoms in patients with thrombotic thrombocytopenic purpura: what are they and when do they occur?. Transfusion.

[4] Wiernek SL, Jiang B, Gustafson GM, Dai X (2018). Cardiac implications of thrombotic thrombocytopenic purpura. World J Cardiol.
